# A Fast Density-Based Clustering Algorithm for Real-Time Internet of Things Stream

**DOI:** 10.1155/2014/926020

**Published:** 2014-06-19

**Authors:** Amineh Amini, Hadi Saboohi, Teh Ying Wah, Tutut Herawan

**Affiliations:** Department of Information System, Faculty of Computer Science and Information Technology, University of Malaya, 50603 Kuala Lumpur, Malaysia

## Abstract

Data streams are continuously generated over time from Internet of Things (IoT) devices. The faster all of this data is analyzed, its hidden trends and patterns discovered, and new strategies created, the faster action can be taken, creating greater value for organizations. Density-based method is a prominent class in clustering data streams. It has the ability to detect arbitrary shape clusters, to handle outlier, and it does not need the number of clusters in advance. Therefore, density-based clustering algorithm is a proper choice for clustering IoT streams. Recently, several density-based algorithms have been proposed for clustering data streams. However, density-based clustering in limited time is still a challenging issue. In this paper, we propose a density-based clustering algorithm for IoT streams. The method has fast processing time to be applicable in real-time application of IoT devices. Experimental results show that the proposed approach obtains high quality results with low computation time on real and synthetic datasets.

## 1. Introduction

**Figure 1:**
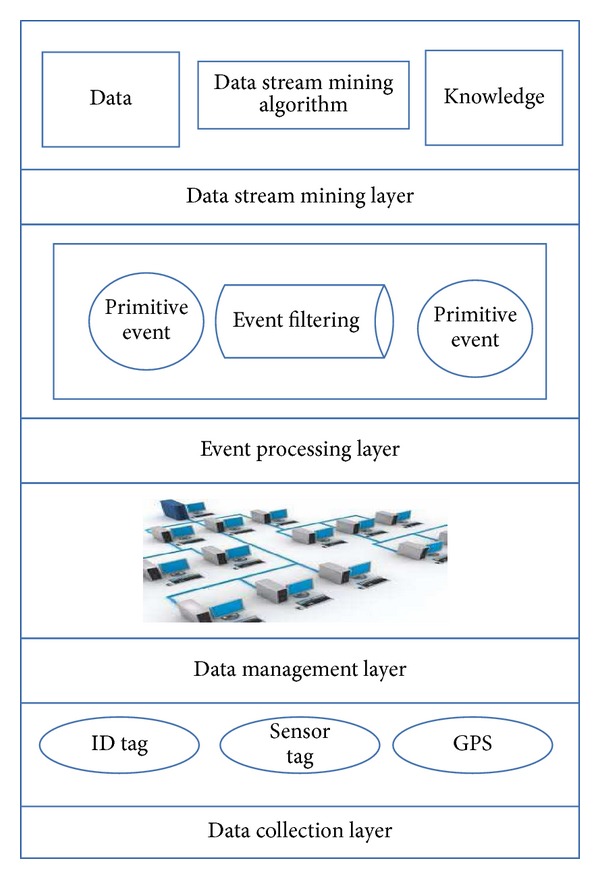
Multilayer data stream mining model for Internet of Things (adopted from [4]).

Using RFID and conventional sensors in the base of the data collection mechanisms in Internet of Things (IoT) makes the volume of the collected data intensively large. In many cases, the communications and data transfers between the objects are required to enable smart analytics. Such communications and transfers require both bandwidth and energy consumption, which are usually limited resources in real scenarios. Furthermore, the analytics required for such applications is often real-time, and therefore it requires the design of methods which can provide real-time insights [1–3]. Data mining techniques are very useful for this kind of analytics. However, since the generated data is considered as stream, we modify the multilayer data mining model for Internet of Things (IoT) from [4] to a multilayer data stream mining model for IoT. The model is illustrated in Figure 1.

Mining data stream is relatively a new area of research in the data mining community. It became more prominent in many applications such as monitoring environmental sensors, social network analysis, real-time detection of anomalies in computer network traffic, and web searches [5, 6].

Clustering is a remarkable task in mining data stream [6]. However, data stream clustering needs some important requirements due to data streams' characteristics such as clustering in limited memory and time with single pass over the evolving data streams and also handling noisy data [7–9].

There are different methods for clustering data streams. In clustering methods, data are categorized based on the similarities among objects. The similarity is determined based on distance or density [5]. The distance-based method [10] leads to form only spherical shapes. On the other hand, density-based method [11] has the ability to detect any shape cluster and they are useful for identifying the noise.

In the last few years, many proposals to extend density-based clustering for data stream have been presented [12]. Density-based data stream clusterings are mainly grouped as density grid-based method and density microclustering method.

The density grid-based clustering [13] quantizes the data space into a number of density grids that form a grid structure on which all of the operations for clustering are performed. The main advantage of the approach is its fast processing time, which is independent of the number of data points, yet dependent on only the number of cells. However, they may have lower quality and accuracy of the clusters despite the fast processing time of the technique [5]. Some of density grid-based clustering algorithms are D-Stream [14], MR-Stream [9], and ExCC [15].

On the other hand, in density-based microclustering [16], microclusters keep summary information about data and clustering is performed on this synopsis information. Microcluster [10] is a temporal extension of cluster feature (CF), that is, a summarization triple maintained about a cluster. Density-based microclustering methods keep summary of clusters in microclusters and form final clusters from them. They have better quality compared to grid-based ones but need more computation time. Some of the density microclustering algorithms include DenStream [14], FlockStream [17], and SOStream [18].

To mitigate the problem of density microclustering methods, we propose a hybrid density-based method for clustering evolving data streams. Our proposed method uses the advantages of both density grid-based and microclustering methods. We refer to our algorithm as HDC-Stream (hybrid density-based clustering for data stream). HDC-Stream has three steps: in step one, the new data point is either mapped to the gird or merged to an existing minicluster. Minicluster is a concept similar to microcluster which is formed from a grid cell. Second step prunes miniclusters and grids in each pruning time. Last step forms the final clusters from the pruned miniclusters using a modified DBSCAN algorithm.

The main contributions of HDC-Stream are summarized as follows.

1. In HDC-Stream, instead of searching list of outlier microclusters to find the suitable one, it maps the new data point into the grid cell which saves computation time. This reduces the number of comparisons from *o*(mi) in finding outlier microclusters to *o*(1) which is the mapping time. mi is the number of miniclusters.
2. In HDC-Stream, instead of forming a new microcluster for a new data point, which is not placed in any existing microcluster and may be a seed of outlier, the new data point is mapped and kept in the grid until the grid density reaches a predefined threshold. In this case, it is converted to a minicluster.
3. The experimental results also show that it outperforms two of the well-known existing density microclustering and density grid-based clustering methods in terms of quality and execution time. Furthermore, the experimental results show that HDC-Stream obtains clusters of high quality even when the noise is present.

The remainder of this paper is organized as follows: Section 2 surveys related work.  Section 3 introduces basic definitions. In Section 4, we explain in detail the HDC-Stream algorithm. We analyze the HDC-Stream algorithm using synthetic and real datasets in Section 5.  Section 6 discusses the advantages of the proposed method. We conclude the paper in Section 7.

## 2. Related Work

Clustering is an important task in data stream mining. Recently, a plenty of clustering algorithms have been developed for data streams. These clustering algorithms can be generally grouped into the four following main categories [5].

A partitioning-based clustering algorithm tries to find the best partitioning for data points in which intraclass similarity is maximum and interclass similarity is minimum. Two of the well-known extensions of *k*-means [19, 20] on data streams are STREAM [7] and CluStream [10]. Hierarchical clustering algorithms work by decomposing data objects into a tree of clusters. BIRCH [10] and ClusTree [8] are examples of hierarchical clustering family. Grid-based clustering is independent of the distribution of data objects. In fact, it partitions the data space into a number of cells, which forms the grids. Grid-based clustering has fast processing time since it is not dependent on the number of data objects. D-Stream [14], MR-Stream [9], and ExCC [15] are grid-based clusterings over data stream.

Density-based clustering algorithms have been developed to discover clusters with arbitrary shapes. They find clusters based on the dense areas in a shape. If two points are close enough and the region around them is dense, then these two data points join and contribute to construction of a cluster. DBSCAN [21], OPTICS [22], and DENCLUE [23] are examples of this approach.

Due to data streams' characteristics, the traditional density-based clustering is not applicable. Recently, many density-based clustering algorithms are extended for data streams. The main idea in these algorithms is using density-based method in the clustering process and at the same time overcoming the constraints, which are put by data stream's nature. Density-based clustering algorithms are categorized into two broad groups called density microclustering and density grid-based clustering algorithms. A comprehensive survey on density-based clustering algorithm on data stream is presented in [12].

DenStream [24] is a density microclustering algorithm for evolving data stream. The algorithm extends the microcluster [10] concept and introduces the outlier and potential microclusters to distinguish between outliers and the real data. It has online and offline phases. In the online phase, the microclusters are formed and the offline phase performs macroclustering on the microclusters. FlockStream [17] is an extension of DenStream using a bioinspired model. It is based on flocking model [25] in which agents are microclusters and they work independently but form clusters together. It considers an agent for each data point which is mapped in the virtual space. Agents move in their predefined visibility range for a fixed time. If they visit another agent, they join to form a cluster in case they are similar to each other. It merges the online and offline phases since the agents form the clusters at any time. In FlockStream, searching for the similar agents is a time consuming process. SOStream (self-organizing density-based clustering over data stream) [18] detects structures within fast evolving data streams by automatically adapting the threshold for density-based clustering. SOStream dynamically creates, merges, and removes clusters in an online manner. It uses competitive learning as introduced for SOMs (self-organizing maps) [26] which is a time consuming method for clustering data stream. Density microclusterings are effective in terms of quality and they can capture the evolution of clusters effectively. However, they have high computation time in finding suitable microclusters.

The other important category is density grid-based method. D-Stream [27] is a density grid-based clustering algorithm in which the data points are mapped to the corresponding grids and the grids are clustered based on their density. It adjusts the clusters in real-time and captures the evolving behavior of data streams and has techniques for handling the outliers. MR-Stream [9] is another clustering algorithm which has the ability to cluster data stream at multiple resolutions. The algorithm partitions the data space into cells and a tree-like data structure which keeps the space partitioning. The tree data structure keeps the data clustering in different resolutions. Each node has the summary information about its parent and children. The algorithm improves the performance of clustering by determining the right time to generate the clusters. D-Stream and MR-Stream algorithms cannot work properly for high dimensional data stream [12]. ExCC (exclusive and complete clustering) [15] is a density grid-based clustering for heterogeneous data stream. The algorithm maps the numerical attributes to the grid and the categorical attributes are assigned granularities according to distinct values in respective domain sets. ExCC introduces fast and slow stream based on the average arrival time of the data points in the data stream. The algorithm detects noise in the offline phase using wait and watch policy. For detecting real outliers, it keeps the data points in the hold queue, which is kept separately for each dimension. The hold queue strategy needs more memory and processing time since it is defined for each dimension. Density grid-based clustering has lower quality since it depends on the granularity of clustering. On the other hand, they can handle the outlier effectively. The computation time is high for high dimensional data.

## 3. Basic Definitions of HDC-Stream

**Definition 1 (*ϵ*-neighborhood of a point).**

The neighborhood is within a radius of *ϵ*. Neighborhood of point *p* is denoted by *N*_*ϵ*_(*p*):

(1) $$\begin{array}{c} N_{\epsilon }\left(p\right)=\left\{q\in D\mathrm{dist}\left(p,q\right)\leq \epsilon \right\}, \end{array}$$

where dist⁡(*p*, *q*) is an Euclidean distance between *p* and *q*.

**Definition 2 (MinPts).**

MinPts is the minimum number of data points around a data point *p* in the *ϵ*-neighborhood of *p*.

**Definition 3 (data point weight value).**

For each data point in the data stream, we consider a weight which decreases over time. The initial value of data point is 1. The weight of data point *x* (with *d* dimensions) in time *t*_*c*_ is defined based on the weight in *t*_*p*_ as follows (*t*_*c*_ > *t*_*p*_):

(2) $$\begin{array}{c} w\left(x,t_{c}\right)=w\left(x,t_{p}\right)f\left(t_{c}-t_{p}\right), \end{array}$$

where function *f* is a fading function. The fading function [28] that we use in HDC-Stream is defined as *f*(*t*) = 2^−*λt*^, where *λ* > 0.

**Definition 4 (grid weight).**

For a grid *g* at current time *t*_*c*_, the grid weight is defined based on sum of data points' weights which are mapped to it:

(3) $$\begin{array}{c} w\left(g,t_{c}\right)=\underset{x\in g}{\sum }2^{-\lambda (t_{c}-t_{x})}. \end{array}$$

According to the work presented in [27], we update the grid weight in *t*_*c*_ with the last updated value *t*_*p*_ as follows:

(4) $$\begin{array}{c} w_{g}\left(t_{c},x\right)=2^{-\lambda (t_{c}-t_{p})}*w_{g}\left(t_{p}\right)+1. \end{array}$$

The total weight of all the grids in data space *S* is *w*(*S*, *t*) = ∑_*x*∈*S*(*t*)_ *w*(*x*, *t*) which is less than 1/(1 − 2^−*λ*^). Moreover, we have

(5) $$\begin{array}{c} \underset{t\to \infty }{\lim}\underset{x\in S\left(t\right)}{\sum }w\left(x,t\right)=\frac{1}{1-2^{-\lambda }}. \end{array}$$

It means that sum of all data points' weights has an upper bound of 1/(1 − 2^−*λ*^). The number of grids equals *N*, which is *N* = ∏_*i*=1_^*d*^ *P*_*i*_, and every *i*th dimension is divided into *P*_*i*_ partitions. Therefore, the average density of each grid is 1/*N*(1 − 2^−*λ*^).

**Definition 5 (core point).**

It is defined as an object for which its overall weight of all *ϵ*-neighborhood data points is at least a value 1/*N*(1 − *λ*).

**Definition 6 (dense grid).**

At time *t*, for a grid *g*, we call it a dense grid if *w*_*g*_(*t*) ≥ *α*/*N*(1 − 2^−*λ*^).

**Definition 7 (sparse grid).**

At time *t*, for a grid *g*, we call it a sparse grid if *w*_*g*_(*t*) < *α*/*N*(1 − 2^−*λ*^).

Because the overall weight cannot be more than 1/(1 − *λ*), *α* is a controlling threshold.

**Definition 8 (minicluster (MIC)).**

A MIC at time *t* is defined as MIC(*w*, *c*, *r*) for a group of very close data points *p*_*i*__1_,…, *p*_*i*__*n*_ with timestamps *T*_*i*__1_,…, *T*_*i*__*n*_ as follows:

(6) $$\begin{array}{c} w_{\text{MIC}}=w_{g}\left(t\right),\;\;w_{g}\left(t\right)\geq \frac{\alpha }{N\left(1-2^{-\lambda }\right)},\\ \text{cente}{\text{r}}_{\text{MIC}}=\frac{\sum_{j=1}^{n}2^{-\lambda (t-{T_{i}}_{j})}\left({p_{i}}_{j}\right)}{w_{\text{MIC}}},\\ \text{radiu}{\text{s}}_{\text{MIC}}=\frac{\sum_{j=1}^{n}2^{-\lambda (t-{T_{i}}_{j})}\text{distance}\left({p_{i}}_{j},c_{\text{MIC}}\right)}{w_{\text{MIC}}}, \end{array}$$

where distance(center_MIC_, *p*_*ij*_) is an Euclidean distance between the center of minicluster and the data points in that grid cell.

**Definition 9 (grid synopsis).**

Is a tuple GS(*n*_*g*_, *t*_*p*_, *w*_*g*_) where *n*_*g*_ is the number of data points, *t*_*p*_ is the last timestamp and *w*_*g*_ is the grid weight.

**Definition 10 (outlier weight threshold (OWT)).**

This threshold is considered for the sparse grids which do not receive any data for long. In fact, these grids do not have any chance to be converted to dense grids and consequently to MIC. If the grid weight is less than this threshold, it can safely be deleted from the grid list (in the outlier buffer) [14]. If the last updated time of grid *g* is *t*_*p*_, then, at current time *t*_*c*_, the outlier weight threshold is defined as follows (*t*_*c*_ > *t*_*p*_):

(7) $$\begin{array}{c} \text{OWT}\left(t_{c},t_{p}\right)=\frac{\alpha }{N}\overset{t_{c}-t_{p}}{\underset{i=0}{\sum }}2^{-\lambda i}=\frac{\alpha \left(1-2^{-\lambda (t_{c}-t_{p}+1)}\right)}{N\left(1-2^{-\lambda (t_{p})}\right)}. \end{array}$$

**Definition 11 (pruning time).**

We check all MICs' weights as well as the weights of all grid cells in a time we call it *t*_*pt*_. *t*_*pt*_ is the minimum time for a MIC in timestamp *t*_1_ to be converted to an outlier in *t*_2_ (*t*_2_ > *t*_1_) which is described as follows:

**Lemma 12 .**

(8) $$\begin{array}{c} t_{pt}=\log_{\lambda }^{\alpha /\left(\alpha -N(1-2^{-\lambda })\right)}. \end{array}$$

**Proof**

(9) $$\begin{array}{c} w_{\text{MIC}}\left(t_{2}\right)=2^{-\lambda (t_{2}-t_{1})}*w_{\text{MIC}}\left(t_{1}\right)+1,\\ \frac{\alpha }{N\left(1-2^{-\lambda }\right)}=2^{-\lambda (t_{2}-t_{1})}\frac{\alpha }{N\left(1-2^{-\lambda }\right)}+1,\;\;t_{pt}=t_{2}-t_{1},\\ t_{pt}=\log_{\lambda }^{\alpha /\left(\alpha -N(1-2^{-\lambda })\right)}. \end{array}$$

## 4. HDC-Stream Algorithm

**Figure 2:**
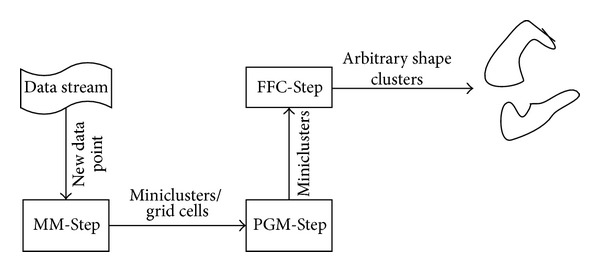
Overall view of HDC-Stream algorithm.

HDC-Stream is a hybrid density-based clustering algorithm for evolving data streams. The overall architecture of HDC-Stream algorithm is outlined in Algorithm 1. It has an online-offline component. For a data stream, at each timestamp, the online component of HDC-Stream continuously reads a new data record and either adds it to an existing minicluster or maps it to the grid. In pruning time, HDC-Stream periodically removes real outliers. The offline component generates the final clusters on demand by the user. The procedure adopted in this algorithm is divided into three steps as follows. The steps are also illustrated in Figure 2.

1. Merging or papping (MM-Step): the new data point is added to an existing minicluster or mapped to the grid (lines 5–18 of Algorithm 1).
2. Pruning grids and miniclusters (PGM-Step): the grids cells as well as miniclusters' weights are periodically checked in pruning time. The periods are defined based on the minimum time for a minicluster to be converted to an outlier. The grids and the miniclusters with the weights less than a threshold are discarded, and the memory space is released (lines 19–33 of Algorithm 1).
3. Forming final clusters (FFC-Step): final clusters are formed based on miniclusters which are pruned. Each minicluster is clustered as a virtual point using a modified DBSCAN (lines 34–36 of Algorithm 1).

The steps are explained as follows.

### 4.1. MM-Step of HDC-Stream

**Figure 3:**
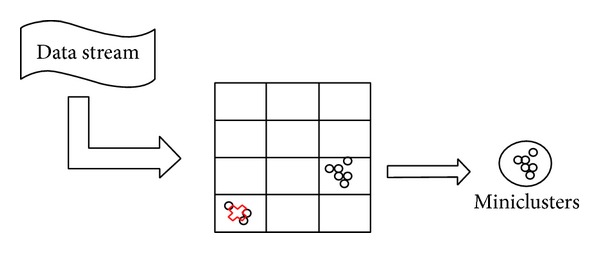
MM-Step of HDC-Stream algorithm.

When a new data point arrives (Figure 3), we get the following.

- HDC-Stream finds the nearest MIC to the new data point.
- If the new data point's distance to the nearest MIC is less than *r*_MIC_, it will be added to that particular MIC.
- Otherwise, the data point has to be mapped into the grid in the outlier buffer.
  - If the number of data points in grid *n*_*g*_ reaches MinPts, then we check the grid weight *w*_*g*_.
    - If the grid weight *w*_*g*_ is higher than the dense grid threshold, then we form a new MIC out of the data points in this grid.
    - The related grid *g* of the new MIC is discarded from the grid list.

### 4.2. PGM-Step of HDC-Stream

For each MIC, if no new point is added, its weight will gradually decay. Furthermore, there are some grids which do not receive data points for a long time and become sporadic. These kinds of MIC and grid cells should be removed from the miniclusters and the grid list, respectively. The decision for removing grids and miniclusters is made based on a comparison of their weights and a specified threshold. Therefore, PGM-Step is performed in each *t*_*pt*_ which is defined in Definition 11.

### 4.3. FCC-Step of HDC-Stream

When a clustering request arrives, a variant of DBSCAN algorithm is applied on the set of the online maintained miniclusters to get the clustering result. Each minicluster MIC is considered as a virtual point located at the center of MIC with the weight *w*_MIC_. We adopt the concept of density connectivity from [21], in order to determine the final clusters. All the density-connected MICs form a cluster. The variant of DBSCAN algorithm includes two parameters: *ϵ* and MinPts.

**Definition 13 (directly density-reachable).**

A MIC_*p*_ is directly density-reachable from a MIC_*q*_ with respect to *ϵ* and MinPts if dist⁡(Center_MIC_*p*__, Center_MIC_*q*__) < *r*_MIC_*p*__ + *r*_MIC_*q*__. Dist(Center_MIC_*p*__, Center_MIC_*q*__) is the Euclidean distance between the centers of MIC_*p*_ and MIC_*q*_.

**Definition 14 (density-reachable).**

A MIC_*p*_ is density-reachable from a MIC_*q*_ with respect to *ϵ* and MinPts if there is a chain of miniclusters MIC_1_,…, MIC_*n*_, such that MIC_1_ = MIC_*q*_ and MIC_*n*_ = MIC_*p*_ (MIC_*p*__*i*+1_ is directly density reachable from MIC_*p*__*i*_).

**Definition 15 (density-connected).**

A MIC_*p*_ is density-connected to a MIC_*q*_ with respect to *ϵ* and MinPts if there is a minicluster MIC_*k*_ such that both MIC_*p*_ and MIC_*q*_ are density-reachable from MIC_*k*_ with respect to *ϵ* and MinPts.

## 5. Experimental Evaluation

In this section, we present the evaluation of HDC-Stream with respect to two existing well-known methods DenStream and D-Stream. We have implemented HDC-Stream as well as the comparative methods in Java. All experiments were conducted on a 2.5 GHz machine with 4 GB memory, running on Mac OS X. In this section, firstly, we describe the datasets and then evaluation measures used for the evaluation of the HDC-Stream algorithm. Detailed experiments on real and synthetic datasets are discussed as well.

### 5.1. Datasets

**Figure 4:**
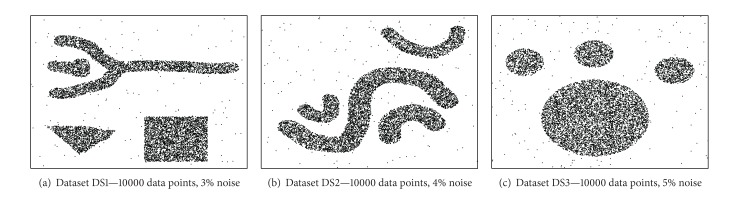
Synthetic datasets.

For evaluation purposes, the clustering quality, scalability, and sensitivity of the HDC-Stream algorithm on both real and synthetic datasets are used. We generated three synthetic datasets DS1, DS2, and DS3 which are depicted in Figures 4(a), 4(b), and 4(c), respectively. DS1 has 10000 data points with 5% noise. DS2 has 10000 data points with 4% noise, and DS3 has 10000 data points with 5% noise. Eventually, we generated an evolving data stream (EDS) by randomly selecting one of the datasets (DS1, DS2, and DS3) 10 times. For each iteration, the chosen dataset forms a 10000-point part of the data stream, so the total length of the evolving data stream is 100000.

The real dataset used is KDD CUP99 Network Intrusion Detection dataset (all 34 continuous attributes out of the total 42 available attributes are used) [29]. The dataset comes from the 1998 DARPA Intrusion Detection. It contains training data consisting of 7 weeks of network-based intrusions inserted in the normal data and 2 weeks of network-based intrusions and normal data for a total of 4,999,000 connection records described by 42 characteristics. KDD CUP99 has been used in [14, 17, 24, 27] and it is converted into data stream by taking the data input order as the order of streaming.

### 5.2. Evaluation Metrics

Cluster validity is an important issue in cluster analysis. Its objective is to assess clustering results of the proposed algorithm by comparing existing well-known clustering algorithms. In the following, we adopt two popular measures, purity and normalized mutual information (NMI), in order to evaluate the quality of HDC-Stream.

#### 5.2.1. Purity

The clustering quality is evaluated by the average purity of clusters which is defined as follows:

(10) $$\begin{array}{c} \text{purity}=\frac{\sum_{i=1}^{K}\left(\left|C_{i}^{d}\right|/\left|C_{i}\right|\right)}{K}*100\%, \end{array}$$

where *K* is  number of clusters, |*C*_*i*_^*d*^| is the number of points with the dominant class label in cluster *i*, and |*C*_*i*_| is the number of points in cluster *i*. The purity is calculated only for the points arriving in a predefined window (*H*), since the weight of points diminishes continuously.

#### 5.2.2. Normalized Mutual Information (NMI)

The normalized mutual information (NMI) is a well-known information theoretic measure that assesses how similar two clusterings are. Given the true clustering *A* = {*A*_1_,…, *A*_*k*_} and the grouping *B* = {*B*_1_,…, *B*_*h*_} obtained by a clustering method, let *C* be the confusion matrix whose element *C*_*ij*_ is the number of records of cluster *i* of *A* that are also in the cluster *j* of *B*. The normalized mutual information, NMI(*A*, *B*), is defined as

(11) $$\begin{array}{c} \text{NMI}\left(A,B\right)=\frac{-2\sum_{i=1}^{c_{A}}\sum_{j=1}^{c_{B}}C_{ij}\log\left(c_{ij}N/C_{i}\cdot C_{j}\right)}{\sum_{i=1}^{c_{A}}C_{i}\log\left(C_{i}/N\right)+\sum_{j=1}^{c_{B}}C_{j}\log\left(C_{j}/N\right)}, \end{array}$$

where *c*_*A*_(*c*_*B*_) is the number of groups in the partition *A*(*B*), *C*_*i*_(*C*_*j*_) is the sum of the elements of *C* in row *i* (column *j*), and *N* is the number of data points. If *A* = *B*, NMI(*A*, *B*) = 1, and if *A* and *B* are completely different, NMI(*A*, *B*) = 0.

The parameters of HDC-Stream adopt the following settings: decay factor *λ* = 0.25, minimum number of points MinPts = 30, and *α* = 0.8. The parameters for DenStream and D-Stream are chosen to be the same as those adopted in [24] and [14], respectively.

### 5.3. Evaluation of HDC-Stream on Synthetic Datasets

**Figure 5:**
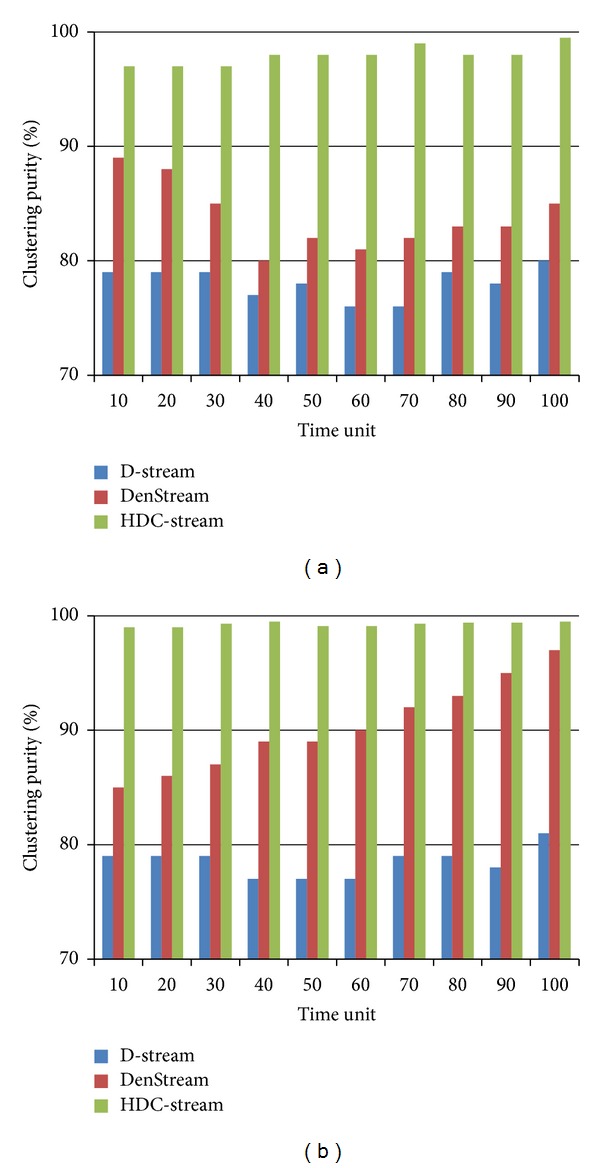
Cluster purity of HDC-Stream for EDS with (a) horizon = 1 and stream speed = 2000 and (b) horizon = 5 and stream speed = 2000.

Figure 5 shows the purity results of HDC-Stream compared to DenStream and D-Stream on EDS data stream. In Figure 5(a), the stream speed is set to 2000 points per time unit and horizon *H* = 1. HDC-Stream shows a good clustering quality. Its clustering purity is higher than 97%. We also set the stream speed at 2000 points per time unit and horizon *H* = 10 for EDS.  Figure 5(b) shows similar results too. We conclude that HDC-Stream achieves much higher clustering quality than DenStream and D-Stream in two different horizons. For example, in horizon *H* = 1, time unit 50, HDC-Stream has 98% while DenStream and D-Stream have purity values as 82% and 78%, respectively.

**Figure 6:**
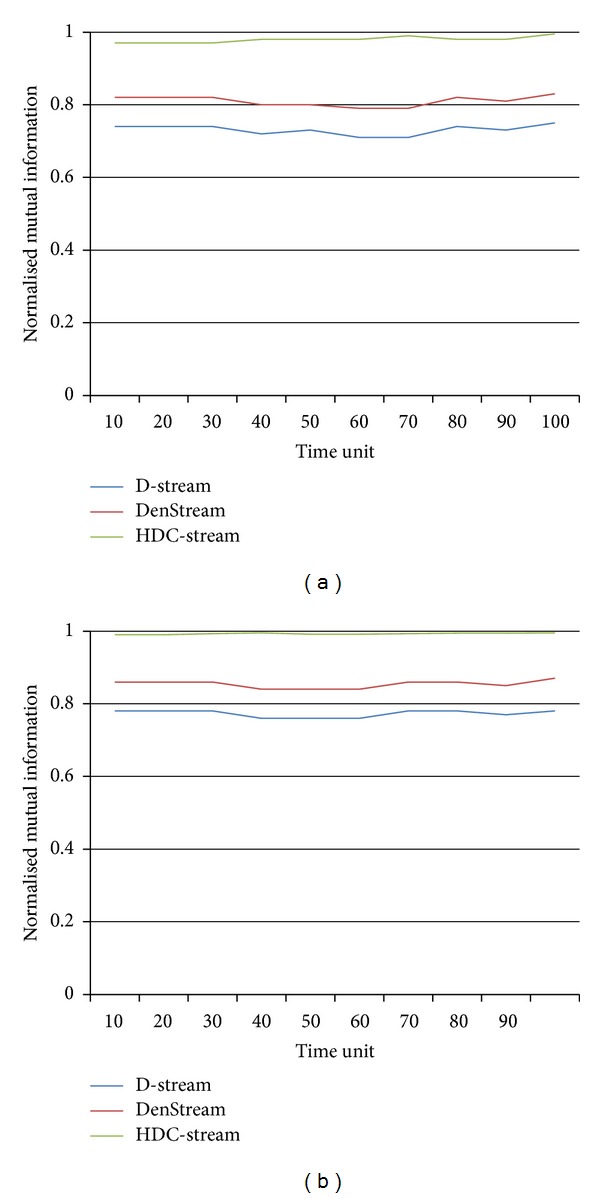
Normalised mutual information of HDC-Stream for EDS with (a) horizon = 1 and stream speed = 2000 and (b) horizon = 5 and stream speed = 2000.

The same is observed from the normalized mutual information aspect. In fact, Figure 6 shows the NMI values obtained by three methods. We repeated the experiments with the same horizon and stream speed (Figures 6(a) and 6(b)). The results show a noticeable high NMI score for HDC-Stream. In fact, its value approaches 1 for both horizons. It also proves that DenStream has better NMI compared to D-Stream.

We noted very good clustering quality of HDC-Stream, D-Stream, and DenStream when no noise is present in the dataset. In fact, purity values are always higher than 98% and all methods are insensitive to the horizon length.

### 5.4. Evaluation of HDC-Stream for Real Datasets

**Figure 7:**
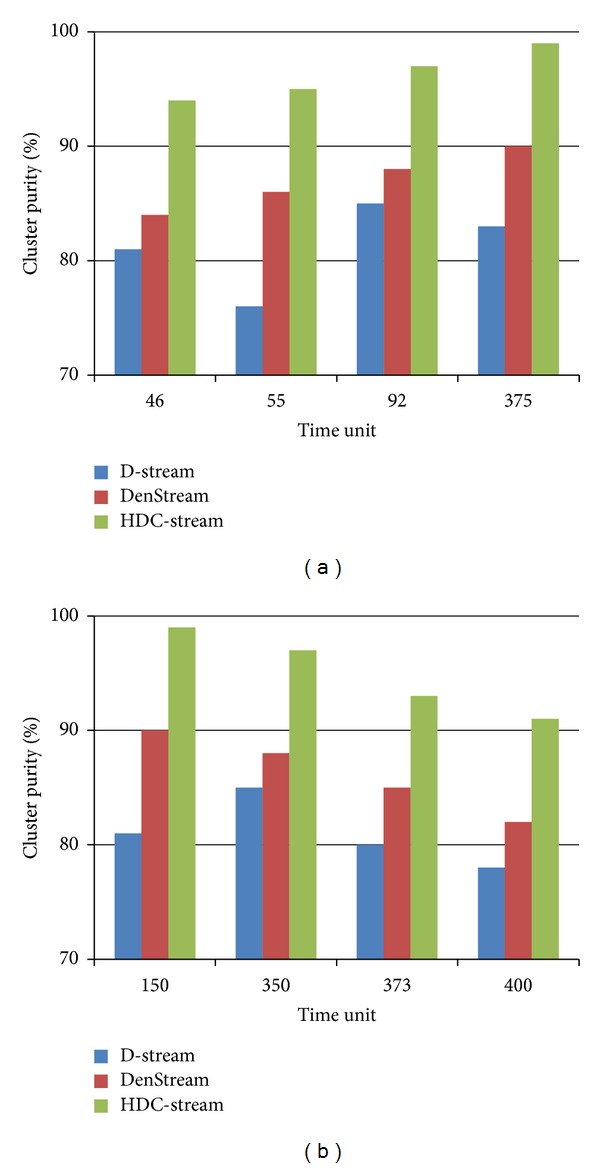
Cluster purity of HDC-Stream for Network Intrusion Detection dataset with (a) horizon = 2 and stream speed = 1000 and (b) horizon = 5 and stream speed = 1000.

The comparison results among HDC-Stream and both DenStream and D-Stream on the Network Intrusion dataset are shown in Figure 7. The evaluation is defined based on the selected time units when the attacks happen on horizons 2 and 5, whereas the stream speed is 1000. For instance, in horizon *H* = 5 and stream speed 1000, there are 99 teardrop attacks, 182 ipsweep attacks, 618 neptune attacks, and 4097 normal connections. HDC-Stream clearly outperforms DenStream and specifically D-Stream. The purity of HDC-Stream is always above 91%. For example, at time 55, the purity of HDC-Stream is about 95% which is higher than both DenStream (86%) and D-Stream (76%).

**Figure 8:**
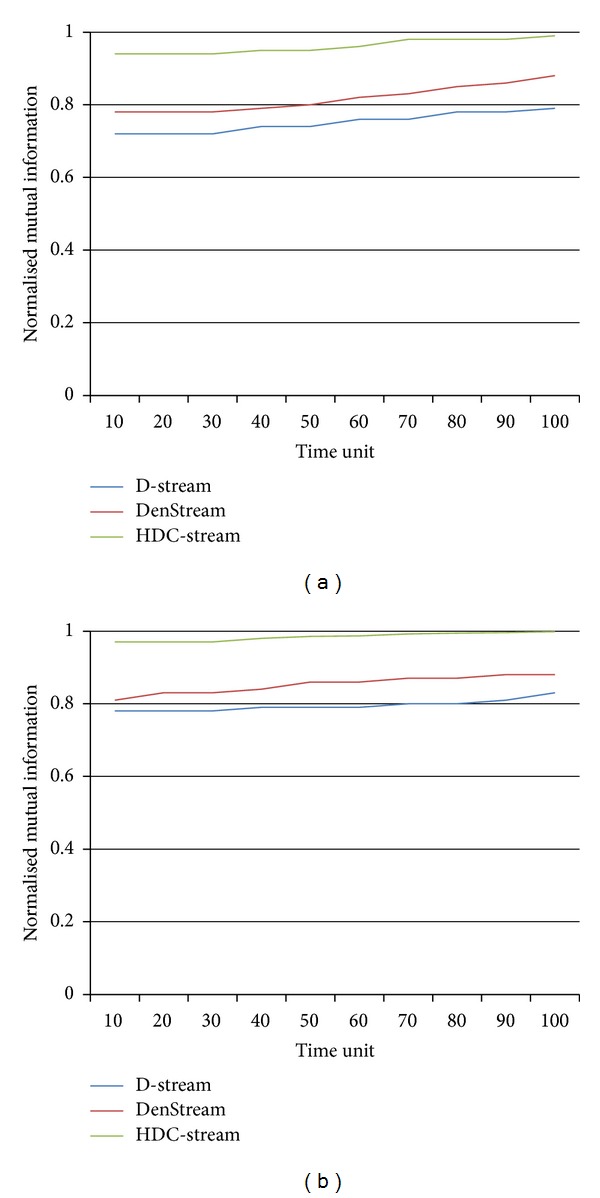
Normalised mutual information of HDC-Stream on Network Intrusion Detection dataset with (a) horizon = 1 and stream speed = 1000, (b) horizon = 5 and stream speed = 1000.

We show the normalized mutual information results on Network Intrusion Detection dataset in Figure 8. The results have been determined by setting the horizon to 1 and 5, whereas the stream speed is 1000 (Figures 8(a) and 8(b)). The values of normalized mutual information for HDC-Stream approach 1 for both horizons. It reveals that HDC-Stream detects the true class labels of data more accurately than DenStream and D-Stream do.

### 5.5. Scalability Results

#### 5.5.1. Execution Time

**Figure 9:**
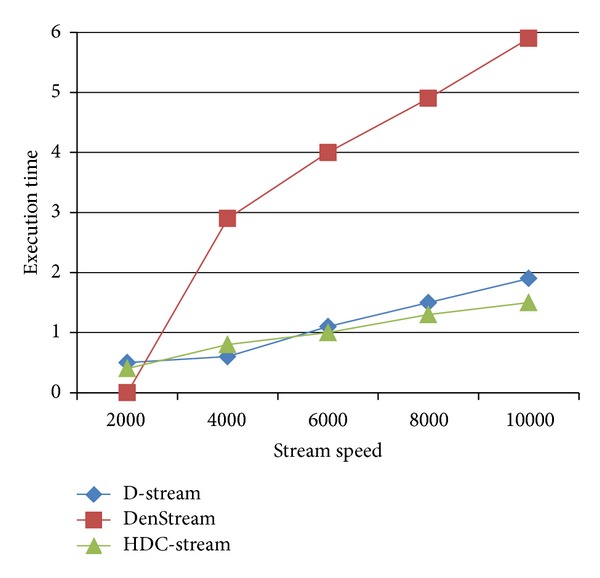
Execution time for increasing stream lengths on Network Intrusion Detection dataset.

The execution time of HDC-Stream is influenced by the number of data points processed at each time unit, that is, the stream speed.  Figure 9 shows the execution time in seconds on Network Intrusion Detection dataset for HDC-Stream compared to DenStream and D-Stream, when the stream speed augments from 1000 to 10,000 data items.

DenStream has higher processing time due to its merging task which is time consuming. HDC-Stream has lower execution time compared to the others. The execution time of other methods increases linearly with respect to the stream speed.

#### 5.5.2. Memory Usage

Memory usage of HDC-Stream is *o*(mi + *g*) which is the total number of miniclusters and grids.

### 5.6. Sensitivity Analysis

**Figure 10:**
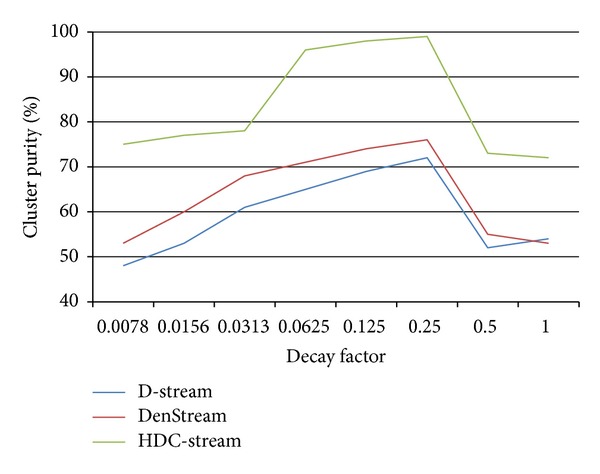
Cluster quality versus decay factor.

An important parameter of HDC-Stream is *λ*. It controls the importance of historical data. We test the quality of clustering on different values of *λ* ranging from 0.0078 to 1 (Figure 10). When *λ* is too small or too large, the clustering quality becomes poor. For example, when *λ* = 0.0078, the purity is about 75%, and, when *λ* = 0.5, the points decay soon after their arrival, and only a small number of recent points contribute to the final results. So the result is not very good. However, the quality of HDC-Stream is still higher than that of DenStream and D-Stream. It is proved that if *λ* varies from 0.0625 to 0.25, the clustering quality is quite good, stable, and always above 96%.

## 6. Discussion

We proposed a hybrid method for clustering evolving data streams which has high quality and low computation time compared to existing methods. The algorithm clusters data streams in three distinctive steps. In existing methods such as DenStream, when a new data point arrives, it takes time to search in two lists of microclusters including potentials and outliers in order to find the suitable microcluster. If it is unable to find a microcluster, DenStream forms a new microcluster for that data point which may be a seed of an outlier, hence leading to a low clustering quality result. However, HDC-Stream only searches in potential list and if it cannot find the suitable microcluster, the data point is mapped to the grid, which keeps the outlier buffer. We reduced the time complexity of clustering algorithm using grid-based clustering. The grid-based method allows us to decrease merging time complexity from *o*(mi) to *o*(1). We implemented the grid list in a 2-3-4 tree data structure which makes search and update faster. The size of the grid list is *o*(log⁡_1/*λ*_ *N*) and the time required for search and update in the grid list is *o*(log⁡log⁡_1/*λ*_ *N*). Consider

(12) $$\begin{array}{c} o\left(\text{MM-Step}\right)=o\left(\text{mi}\right)+o\left(\log_{1/\lambda }N\right)+o\left(1\right),\\ o\left(\text{PGM-step}\right)=o\left(\log_{1/\lambda }N\right)+o\left(\text{mi}\right). \end{array}$$

We reduced the number of comparisons; therefore, time complexity for merging to minicluster list is *o*(mi); in which the number of mi is less than number of microclusters in DenStream, since, in that algorithm, there are two lists to keep potential and outlier microclusters. Furthermore, we increased the clustering quality by forming miniclusters from the data points that are surely not outliers. When the grid density reaches the specified threshold, the data points inside that grid form a minicluster. Therefore, we do not need to form a minicluster for a newly arrived data if it cannot be placed in any minicluster. The quality is also increased since miniclusters are never formed from an outlier.

Finally, the evaluation results prove that using a hybrid method for clustering evolving data streams improves the clustering quality results and reduces the computation time.

## 7. Conclusion

In this paper, we proposed a hybrid density-based clustering algorithm for Internet of Things (IoT) streams. Our hybrid algorithm has three steps in which the new data point is either mapped to grid or merged to an existing minicluster, the outliers are removed, and finally arbitrary shape clusters are formed using miniclusters by a modified DBSCAN. Our method is a hybrid one, which uses density grid-based clustering and density microclustering to improve the computation time and quality. The evaluation results on synthetic and real datasets show that it has high quality with low computation time for merging. However, HDC-Stream is not suitable to be used in distributed environments.

Our future work will focus on the improvement of HDC-Stream as a distributed density-based data stream clustering algorithm.

## Figures and Tables

**Algorithm 1:**
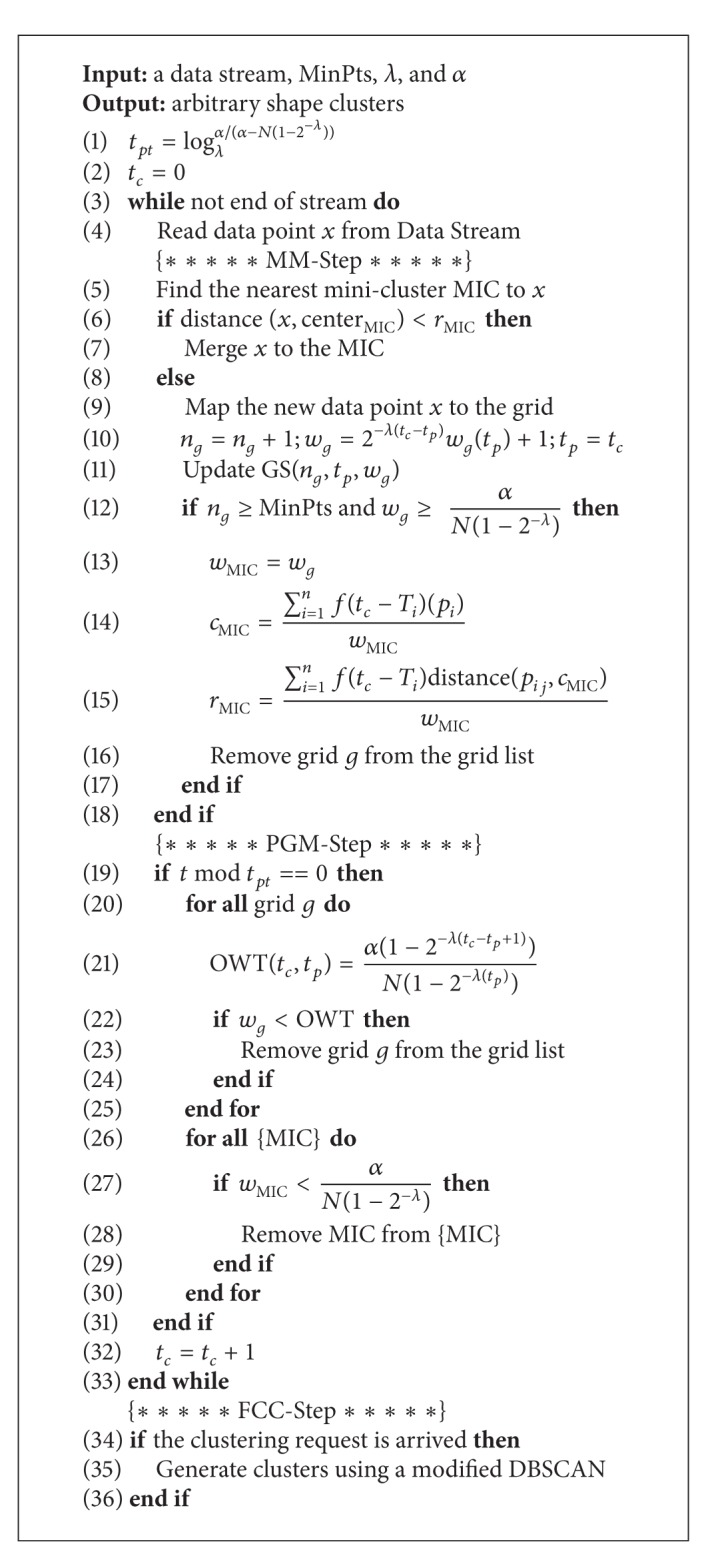
HDC-Stream (DS, MinPts, *λ*, and *α*).
